# A public benchmark for human performance in the detection of focal cortical dysplasia

**DOI:** 10.1002/epi4.70028

**Published:** 2025-04-01

**Authors:** Lennart Walger, Matthias H. Schmitz, Tobias Bauer, David Kügler, Fabiane Schuch, Christophe Arendt, Tobias Baumgartner, Johannes Birkenheier, Valeri Borger, Christoph Endler, Franziska Grau, Christian Immanuel, Markus Kölle, Patrick Kupczyk, Asadeh Lakghomi, Sarah Mackert, Elisabeth Neuhaus, Julia Nordsiek, Anna‐Maria Odenthal, Karmele Olaciregui Dague, Laura Ostermann, Jan Pukropski, Attila Racz, Klaus von der Ropp, Frederic Carsten Schmeel, Felix Schrader, Aileen Sitter, Alexander Unruh‐Pinheiro, Marilia Voigt, Martin Vychopen, Philip von Wedel, Randi von Wrede, Ulrike Attenberger, Hartmut Vatter, Alexandra Philipsen, Albert Becker, Martin Reuter, Elke Hattingen, Alexander Radbruch, Rainer Surges, Theodor Rüber

**Affiliations:** ^1^ Department of Neuroradiology University Hospital Bonn Bonn Germany; ^2^ Department of Epileptology University Hospital Bonn Bonn Germany; ^3^ German Center for Neurodegenerative Diseases (DZNE) Bonn Germany; ^4^ Department of Neuroradiology Goethe University Frankfurt Frankfurt Germany; ^5^ Department of Neurology University Hospital Bonn Bonn Germany; ^6^ Department of Neurosurgery University Hospital Bonn Bonn Germany; ^7^ Department of Diagnostic and Interventional Radiology University Hospital Bonn Bonn Germany; ^8^ Department of Psychiatry and Psychotherapy University Hospital Bonn Bonn Germany; ^9^ Chair of Economic & Social Policy WHU ‐ Otto Beisheim School of Management Vallendar Germany; ^10^ Department of Neuropathology University Hospital Bonn Bonn Germany; ^11^ A. A. Martinos Center for Biomedical Imaging Massachusetts General Hospital Charlestown Massachusetts USA; ^12^ Department of Radiology Harvard Medical School Boston Massachusetts USA; ^13^ Center for Medical Data Usability and Translation University of Bonn Bonn Germany

**Keywords:** artificial intelligence, computer‐aided detection, human performance, reader study

## Abstract

**Objective:**

This study aims to report human performance in the detection of Focal Cortical Dysplasias (FCDs) using an openly available dataset. Additionally, it defines a subset of this data as a “difficult” test set to establish a public baseline benchmark against which new methods for automated FCD detection can be evaluated.

**Methods:**

The performance of 28 human readers with varying levels of expertise in detecting FCDs was originally analyzed using 146 subjects (not all of which are openly available), we analyzed the openly available subset of 85 cases. Performance was measured based on the overlap between predicted regions of interest (ROIs) and ground‐truth lesion masks, using the Dice‐Soerensen coefficient (DSC). The benchmark test set was chosen to consist of 15 subjects most predictive for human performance and 13 subjects identified by at most 3 of the 28 readers.

**Results:**

Expert readers achieved an average detection rate of 68%, compared to 45% for non‐experts and 27% for laypersons. Neuroradiologists detected the highest percentage of lesions (64%), while psychiatrists detected the least (34%). Neurosurgeons had the highest ROI sensitivity (0.70), and psychiatrists had the highest ROI precision (0.78). The benchmark test set revealed an expert detection rate of 49%.

**Significance:**

Reporting human performance in FCD detection provides a critical baseline for assessing the effectiveness of automated detection methods in a clinically relevant context. The defined benchmark test set serves as a useful indicator for evaluating advancements in computer‐aided FCD detection approaches.

**Plain Language Summary:**

Focal cortical dysplasias (FCDs) are malformations of cortical development and one of the most common causes of drug‐resistant focal epilepsy. Once found, FCDs can be neurosurgically resected, which leads to seizure freedom in many cases. However, FCDs are difficult to detect in the visual assessment of magnetic resonance imaging. A myriad of algorithms for automated FCD detection have been developed, but their true clinical value remains unclear since there is no benchmark dataset for evaluation and comparison to human performance. Here, we use human FCD detection performance to define a benchmark dataset with which new methods for automated detection can be evaluated.

**Key points**

- Focal Cortical Dysplasia (FCD) is a congenital brain malformation and a common cause of epilepsy.
- FCDs are often overlooked in the conventional assessment of MRI but have a high chance to result in seizure freedom if successfully resected.
- Automated detection algorithms are being developed, but there is no benchmark dataset for evaluation and comparison to human performance.
- Here, we use human FCD detection performance to define a benchmark dataset with which new methods for automated detection can be evaluated.
- It is hoped that an adequate model evaluation helps the development of novel approaches for automated FCD detection.

## 1 INTRODUCTION

Focal Cortical Dysplasia (FCD) is a localized region of the malformed cerebral cortex and is commonly associated with focal epilepsy.^1^ Surgical treatment of drug‐resistant FCD yields seizure freedom in up to 70% of cases.^2^, ^3^ FCDs can be identified visually in the MRI mainly based on four features: cortical thickening, blurring of the gray–white matter interface, abnormal cortical gyration, and a transmantle sign. They may also appear hyperintense in the FLAIR image. The visual identification of FCD lesions in MRI is linked to successful surgical outcomes,^4^, ^5^ but is only accurate in around two‐thirds of all cases.^6^ In a previous work, we reproduced this success rate by evaluating the performance of human raters with different levels of expertise and in accordance with technical standards^7^ for 146 cases with FCD. Raters had to locate FCDs with a coordinate and a rectangular region of interest (ROI) in the MRI, and we defined quantitative criteria of what it means to “find” an FCD. We showed that human performance depends on the presence of FCD‐specific MRI features; it is especially influenced by FLAIR hyperintensity and cortical thickening. Eighty‐five of the FCD cases were made publicly available in a different work.^8^ The public dataset includes the defaced T1 and FLAIR images, the ground‐truth lesion masks, and other clinical information. One reason to publish such a dataset is to facilitate the comparability of automated FCD detection approaches. However, a baseline of human performance for this specific dataset, which may aid future model evaluation, is currently missing. It is the aim of this work to provide a said baseline by analyzing human performance in lesion detection using the 85 public FCD cases. Additionally, we define a subset of the data as a “difficult” benchmark test set, which contains a representative collection of FCD cases and a collection of hard cases. We hope that defining such a benchmark test set aids the adequate estimation of model performance and spurs the development of novel approaches, even when only public data is available.

## 2 MATERIALS AND METHODS

### 2.1 FCD cohort

The whole cohort used for the rating consisted of 146 patients with FCD and 34 healthy controls. It is previously described in detail.^7^ Here, we only analyze a subset of 85 FCD cases, which are part of the “open presurgery MRI dataset of people with epilepsy and focal cortical dysplasia type II” made publicly available by Schuch et al..^8^ People with epilepsy and FCD (FCD cases) who underwent presurgical evaluation at the University Hospital Bonn from 2006 to 2021 were ascertained. Inclusion criteria were that T1 and FLAIR scans from 3 Tesla MRI scanners were available. The cohort consists of people with a histologically verified or radiologically suspected FCD Type II, with the lesion locations determined by two neuroradiologists and masks annotated by two board‐certified neurologists.^8^ Table 1 summarizes subject‐specific information.

**TABLE 1:** Subject information.

People with epilepsy	
Total [no.]	85
Sex [no.]	
Female	35 (41%)
Male	50 (59%)
Age at MRI‐scan [years]	29 (IQR: 16)
Lesion Volume [ml]	4.98 (IQR: 3.32)
Reported MRI Features [no.]	
Cortical Thickening	66 (78%)
Gray‐White Matter Blurring	60 (71%)
Transmantle Sign	28 (33%)
Abnormal Gyration	15 (18%)
None	1 (1%)
Primary Lesion Location [no.]	
Frontal	52 (61%)
Parietal	20 (24%)
Temporal	5 (6%)
Occipital	6 (7%)
Insula	2 (2%)
Epilepsy surgery [no.]	
Performed	50 (59%)
Not performed	35 (41%)
Histopathology (Palmini) [no.]	
IIa	16 (19%)
IIb	34 (40%)

Abbreviation: IQR, Interquartile range.

### 2.2 Rater cohort

As described in the original publication, voluntary raters were recruited through advertisements on the University Hospitals of Bonn and Frankfurt medical campuses as well as private contacts.^7^ Table 1 shows subject‐specific information. Most importantly, we differentiated raters according to their level of expertise regarding FCD detection. There were three levels of expertise: layperson, that is, someone without a medical degree and not pursuing one, non‐expert physicians, and expert physicians. The latter two levels were differentiated according to the rater's self‐assessed experience with the MRI‐based diagnosis of FCD. Experts included two neuroradiologists and three epileptologists and had an average of 9.1 (interquartile range, IQR: 6.8) years of experience. Non‐experts included three neuroradiologists, five epileptologists, four psychiatrists, four general radiologists, two neurosurgeons, and two neurologists and had an average of 2.1 years of experience (IQR: 4.4). Table 2 shows rater information.

**TABLE 2:** Rater information.

Raters	
Total [no.]	28
Experience in [years]	
General radiology	2.7 (IQR: 3.6)
Neuroradiology	2.4 (IQR: 0.6)
Speciality [no.]	
Epileptology	8 (29%)
Neuroradiology	5 (18%)
Psychiatry	4 (14%)
Radiology	4 (14%)
Neurosurgery	2 (7%)
Neurology	2 (7%)
None	3 (11%)
Raters with different degrees of experience in localizing FCDs (self‐assessed) [no.]	
Layperson	3 (11%), no experience
Non‐expert physician	20 (71%), 2.1 years of experience (IQR: 4.4)
Expert physician	5 (18%), 9.1 years of experience (IQR: 6.8)

Abbreviation: IQR, Interquartile range.

### 2.3 Ethics statement

We confirm that we have read the Journal's position on issues involved in ethical publication and affirm that this report is consistent with those guidelines.

### 2.4 Reading workflow

**FIGURE 1:**
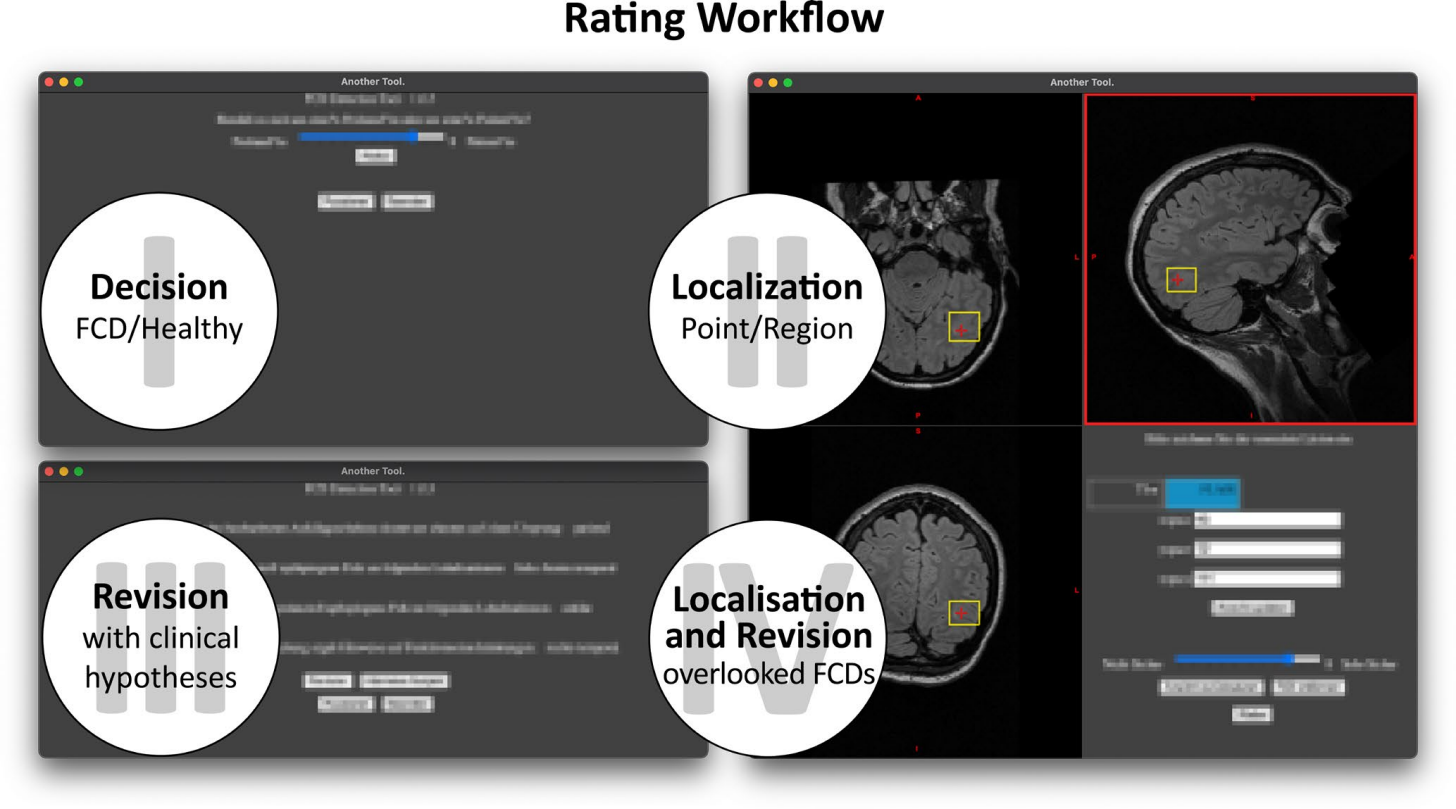
Rating workflow. Raters were guided through the rating process with a specifically developed software based on an open‐source MRI viewer.^9^ After viewing the MRI data in their native diagnostic environment, they first had to choose between FCD and Healthy Control (I)*. If correctly choosing FCD, they proceeded with localizing the suspected lesion via a coordinate (red cross) and rectangular bounding box defining the ROI (yellow) (II). Afterward, raters were shown clinical information and could revise their detection (III). In the end, steps (II) and (III) were repeated for all misclassified FCD cases (IV). *In the original publication, the cohort included healthy controls, while the here analyzed subset does not.

The whole reading process was described in detail in a previous work^7^ and is exemplified in Figure 1. Most importantly, readers had to locate a possible lesion via a single coordinate as well as a three‐dimensional, rectangular bounding box defining the predicted ROI. They also had to provide a confidence score between 0 and 10, indicating how sure they were in their localization. They viewed MRI data on radiological screens; otherwise, the process was guided by a specifically designed software.

### 2.5 Metrics

As described by Walger and colleagues, we measured the voxel‐level overlap between the predicted ROI and an automatically generated bounding box around the ground‐truth annotations via the Dice‐Soerensen coefficient (DSC).^7^ For voxel‐level performance, we additionally report positive predictive value (PPV, precision) and True Positive Rate (TPR, sensitivity) between the automatically defined bounding box around the ground truth and the bounding box/predicted ROI defined by the raters, that is, how much of a lesion was found/how much of the predicted ROI covered the lesion. To determine subject‐level performance, we applied the FCD detection‐specific threshold of 0.22 to the DSC as the criterion for a successful detection.^7^ We analyzed human calibration, that is, how well their indicated localization confidence aligned with the probability of having detected the lesion using the Expected Calibration Error.^10^ As an example, having a detection rate of 70% for predictions with a confidence of 7 (out of 10) would amount to no calibration error.

### 2.6 Statistics

Values are reported as mean [95% confidence interval]. The “test set” was made to contain 28, one third of the total, FCD cases. It consists of two parts, the subjects most predictive for rater performance and the most difficult subjects. We derived the subjects most predictive for rater performance from bootstrapping a Random Forest regression model.^11^ In this procedure, a Random Forest is trained to predict a rater's performance across the whole cohort, based on the subjects they have correctly detected, chosen randomly from a subset of the cohort. We chose each forest to have 1000 single estimators and trained a single forest on 80% (randomly chosen) subjects of the cohort, restricting it to use only 15 subjects. The bootstrapping consisted of rerunning the training 100 times and counting the number of each subject that was chosen. The “most predictive” subjects were the 15 subjects chosen most often. The “most difficult” subjects were found by at most 3 of the 28 raters (~10%).

## 3 RESULTS

### 3.1 Overall rater performance

The DSC score for all raters was 0.24 [0.21, 0.28]. Experts achieved a DSC score of 0.37 [0.38, 0.47]. For PPV, all raters achieved 0.31 [0.30, 0.33], and experts 0.38 [0.35, 0.41], on average. Experts' predictions were significantly more sensitive with an average TPR of 0.50 [0.38, 0.61] compared to 0.29 [0.23, 0.34] for all raters. All raters missed 31% [26, 37] of all FCD cases in the initial run, while experts missed 20% [9, 32]. Overall localization confidence was 5.54 [4.80, 6.29]. Experts were more confident, averaging 7.46 [6.68, 8.25]. Raters had an Expected Calibration Error of 13.74% [11.85, 15.62] and experts of 10.82% [6.43, 15.20]. FCD location did not have an impact on the performance of the raters. Table 3 shows the subject‐level performance depending on the criterion for “finding” an FCD.

**TABLE 3:** Subject‐level human detection rates for the overall cohort and test set. Three thresholds were used for detection: One voxel overlap (OVO): DSC >0.0, applied in other works on FCD detection; standard: DSC >0.7, the computer vision standard^12^; adjusted: DSC >0.22.^7^ All values reported as mean [95% confidence interval].

Rater group	Pinpointing (%)	Detection (%)		
		OVO, 0.0	Standard, 0.7	Adjusted, 0.22
Overall				
Layperson	29 [23, 34]	33 [27, 38]	2 [0, 4]	27 [22, 33]
Non‐expert	48 [46, 51]	54 [52, 56]	3 [2, 4]	45 [42, 47]
Expert	68 [63, 72]	74 [70, 79]	9 [6, 12]	68 [63, 73]
Test set				
Layperson	4 [0, 8]	6 [1, 11]	0 [0, 0]	5 [2, 7]
Non‐expert	29 [25, 33]	33 [29, 37]	1 [0, 2]	26 [19, 32]
Expert	49 [40, 57]	60 [52, 68]	4 [0, 7]	49 [37, 62]

### 3.2 Test set

**FIGURE 2:**
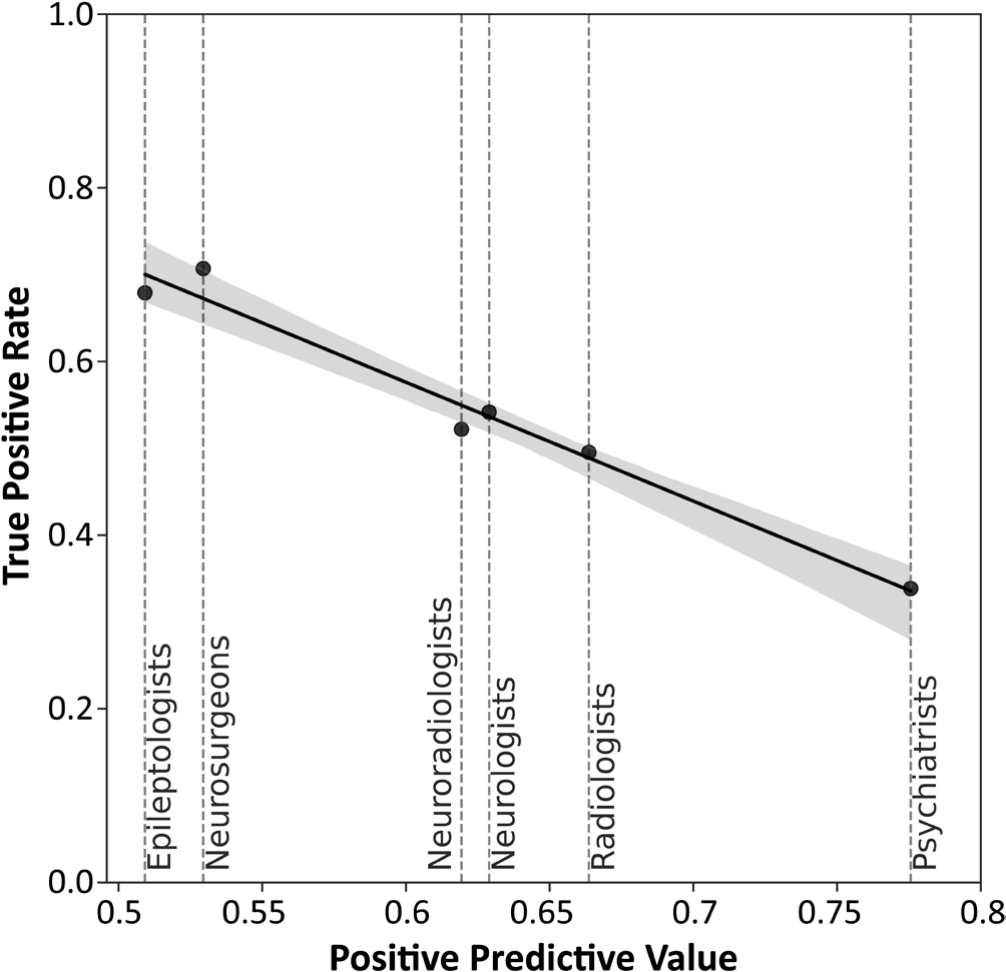
Tradeoff between TPR and PPV for pinpointed lesions across different rater specializations. Shaded area represents the 95% confidence interval.

The test set consists of 28 subjects. Thirteen subjects were found by at most three raters. The bootstrapping procedure yielded a mean absolute error per run of 0.071 [0.068, 0.074]. The resulting random forest regressor trained with the selected subjects achieved a mean absolute error of 0.021 [0.016, 0.026]. All readers achieved an average DSC of 0.14 [0.11, 0.18]; experts achieved an average DSC of 0.26 [0.20, 0.33]. Overall confidence was 4.56 [3.77, 5.34] and 6.24 [4.88, 7.60] for experts. The subject‐level performance in the test set is summarized in Table 3. The IDs for the benchmark test set will be denoted in version 1.0.6 of the dataset, available under doi: 10.18112/openneuro.ds004199.v1.0.6. Figure 2 shows rater predictions for an “easy” and a “hard” case of FCD.

### 3.3 Rater specialization

**FIGURE 3:**
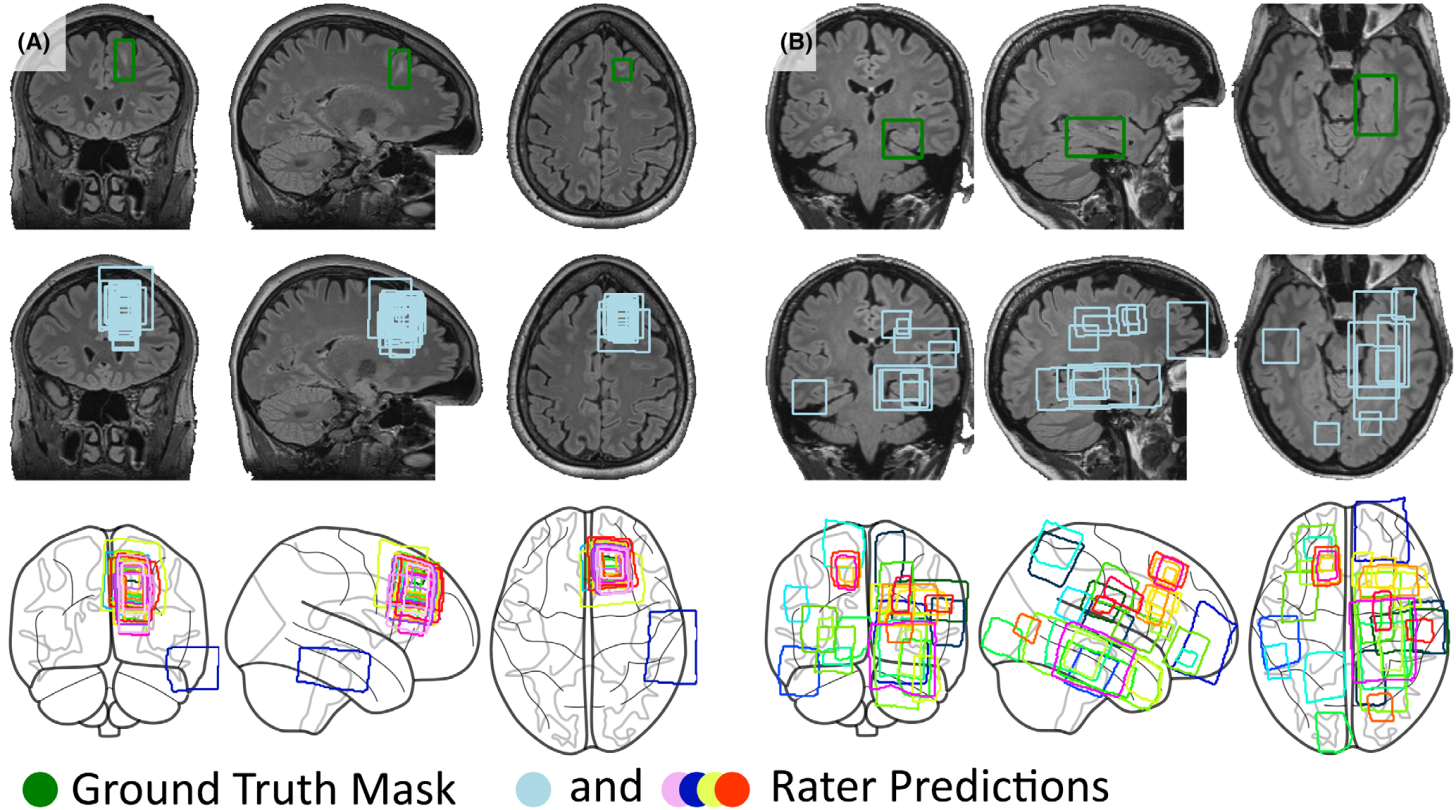
Two examples of rater predictions. One has a detection rate of 96% (A) and the other of 14% (B). Groundtruth lesion mask is denoted by the green rectangle (top), rater bounding boxes/ROI predictions relative to the lesion in light blue (middle) and across the whole brain in various colors (bottom). The annotation may appear non‐rectangular due to registration to MNI space for the purpose of visualization.

Although given the same instructions to draw the ROI “tightly” around the whole area identified as lesional, we found that rating behavior depended on a reader's field of work. Epileptologists' and neurosurgeons' predictions were comparatively large, their predictions having a mean volume of 32 mL [16, 48] and 30 mL [26, 34], respectively. On the other end, psychiatrists drew the smallest ROIs with an average size of 7 mL [6, 8]. The average lesion size for pinpointed lesions did not differ significantly for all reader specializations, with an overall mean volume of 6 mL [6, 7]. Neuroradiologists detected most lesions (64% [59, 69]) and psychiatrists the least (34% [29, 39]). Given a lesion was successfully pinpointed, neurosurgeons achieved the highest TPR with 0.70 [0.65, 0.76] while psychiatrists achieved the lowest with 0.34 [0.29, 0.38]. They, however, achieved the highest PPV of 0.78 [0.74, 0.82] while epileptologists achieved the lowest with 0.50 [0.48, 0.53]. The inverse relationship between TPR and PPV is visualized in Figure 3. The values for all specializations are displayed in Table 4.

**TABLE 4:** Specialization‐specific performance.

	ROI volume (mL)	Pinpointed lesion volume (mL)	TPR (if pinpointed)	PPV (if pinpointed)	Detection rate (%)
Specialization					
Epileptology	32 [16, 48]	7 [6, 8]	0.68 [0.65, 0.71]	0.51 [0.48, 0.53]	51 [48, 55]
Neurosurgery	30 [26, 34]	7 [5, 10]	0.71 [0.65, 0.76]	0.53 [0.47, 0.59]	42 [35, 50]
Neurology	13 [11, 15]	7 [5, 10]	0.54 [0.48, 0.60]	0.63 [0.58, 0.68]	44 [36, 51]
Neuroradiology	13 [12, 15]	6 [5, 7]	0.52 [0.49, 0.55]	0.62 [0.59, 0.65]	64 [59, 69]
Psychiatry	7 [6, 8]	7 [5, 9]	0.34 [0.30, 0.38]	0.78 [0.74, 0.82]	34 [29, 39]
Radiology	13 [11, 16]	7 [5, 8]	0.50 [0.45, 0.54]	0.66 [0.62, 0.70]	43 [38, 48]

*Note*: All values reported as mean [95% confidence interval].

## 4 DISCUSSION

Our study aimed to establish a baseline for human performance in detecting Focal Cortical Dysplasia (FCD) using a publicly available dataset. The results revealed significant variability in detection rates among readers with different levels of expertise. Expert readers demonstrated a higher detection rate of 68%, compared to 45% for non‐experts and 27% for laypersons. Neuroradiologists detected the highest percentage of lesions (64%), whereas psychiatrists detected the least (34%). Neurosurgeons achieved the highest sensitivity (0.70) and psychiatrists the highest precision (0.78). The “difficult” benchmark test set, consisting of 15 cases most predictive of human performance and 13 cases detected by at most three readers, further highlighted these differences, with overall lower detection scores but maintained trends in expertise‐related performance disparities.

Several limitations should be considered when interpreting these results. First, the sample size of 85 FCD cases, though substantial, is relatively small and may not fully represent the variability found in broader clinical settings, especially since the dataset contains only cases with FCD type II. Also, one has to bear in mind, that in clinical practice, clinical notes are considered in the reading of MRI images. These clinical notes were not available for the current/public dataset, however, have been analyzed in the full dataset.^7^ The performance of the experts and non‐experts increased after having seen the clinical notes, whereas the performance of laypersons did not change significantly. Additionally, the reliance on self‐assessed expertise may introduce bias, as individuals' confidence in their abilities might not accurately reflect their true skill levels. The study also did not account for potential variability in the quality of MRI scans, which could influence detection rates. Furthermore, the definition of the “difficult” benchmark test set based on the number of raters successfully detecting the lesion may not represent all the nuances of challenging cases in real‐world scenarios.

The study highlighted substantial differences in performance based on the readers' specialization. Neuroradiologists and neurosurgeons, who interpret MRI images as part of their clinical routine, performed better in terms of detection rates and voxel‐level sensitivity. They marked a comparatively large area, which resulted in more true and false positive voxels. In contrast, psychiatrists, who typically have less experience with MRI‐based lesion identification, showed lower detection rates but higher voxel‐level precision. They marked a comparatively small area, which resulted in fewer true and false positive voxels. This discrepancy underscores the importance of specialization and experience in accurately detecting FCD. It also suggests that training programs for FCD detection should be tailored to the specific needs and backgrounds of different medical professionals to optimize diagnostic accuracy. Automated approaches may help with the standardization of diagnostic imaging, but their effect may also vary depending on who uses them. In the future, the interaction between physicians from varying medical backgrounds and automated approaches needs to be studied. Automated approaches could then also be specifically tailored to better complement human performance.

## 5 CONCLUSION

Defining a human performance baseline for FCD detection is essential for evaluating the effectiveness of automated detection systems. Our results provide a reference point against which new computer‐aided detection (CAD) methods can be evaluated. The “difficult” benchmark test set serves as a valuable tool for assessing the robustness and sensitivity of these systems in challenging cases.

Future work should aim to expand the dataset to include a more diverse and larger cohort of FCD cases, thus improving the generalizability of the results. Additionally, incorporating advanced imaging techniques and multimodal data could enhance detection accuracy. Further research should also explore the impact of targeted training interventions on improving the detection skills of non‐specialist readers, potentially narrowing the performance gap between different professional groups. By continuing to refine and validate CAD systems against robust human benchmarks, we can advance toward more reliable and accurate diagnostic tools for FCD detection in clinical practice.

## FUNDING INFORMATION

No external funding was received for this work.

## CONFLICT OF INTEREST STATEMENT

AR has received fees as a speaker from UCB Pharma and travel support from the Elisabeth und Helmut Uhl Stiftung. UA has received fees as a speaker for Siemens Healthineers and as a clinical consultant for Bayer. AR lectures for Guerbet and Bayer and is part of the Advisory Board for GE, Bracco, and Guerbet. RS has received personal fees as a speaker or for serving on advisory boards from Angelini, Arvelle, Bial, Desitin, Eisai, Jazz Pharmaceuticals Germany GmbH, Janssen‐Cilag GmbH, LivaNova, LivAssured BV, Novartis, Precisis GmbH, Rapport Therapeutics, Tabuk Pharmaceuticals, UCB Pharma, UNEEG, and Zogenix. TR has received fees as a speaker from Eisai. None of the previously mentioned activities were related to the content of this manuscript. The remaining authors have nothing to declare. We confirm that we have read the Journal's position on issues involved in ethical publication and affirm that this report is consistent with those guidelines.

## Data Availability

The data that support the findings of this study are openly available in OpenNeuro at https://openneuro.org/datasets/ds004199/versions/1.0.6.
